# Combined scRNAseq and Bulk RNAseq Analysis to Reveal the Dual Roles of Oxidative Stress-Related Genes in Acute Myeloid Leukemia

**DOI:** 10.1155/2023/5343746

**Published:** 2023-02-09

**Authors:** Jing Qi, Jingyu Lin, Changjiang Wu, Hesheng He, Junping Yao, Youhai Xu, Yuqiong Yang, Yuanfeng Wei, Dongping Huang, Yiming Mao

**Affiliations:** ^1^Department of Hematology, The First Affiliated Hospital of Wannan Medical College, Wuhu, 241001 Anhui, China; ^2^Department of Science & Education, Suzhou Kowloon Hospital, Shanghai Jiao Tong University School of Medicine, Suzhou, 215028 Jiangsu, China; ^3^Department of Intensive Care Unit, Suzhou Kowloon Hospital, Shanghai Jiao Tong University School of Medicine, Suzhou, 215028 Jiangsu, China; ^4^Department of thoracic surgery, Suzhou Kowloon Hospital, Shanghai Jiao Tong University School of Medicine, Suzhou, 215028 Jiangsu, China

## Abstract

**Background:**

Oxidative stress (OS) can either lead to leukemogenesis or induce tumor cell death by inflammation and immune response accompanying the process of OS through chemotherapy. However, previous studies mainly focus on the level of OS state and the salient factors leading to tumorigenesis and progression of acute myeloid leukemia (AML), and nothing has been done to distinguish the OS-related genes with different functions.

**Method:**

First, we downloaded single-cell RNA sequencing (scRNAseq) and bulk RNA sequencing (RNAseq) data from public databases and evaluated the oxidative stress functions between leukemia cells and normal cells by the ssGSEA algorithm. Then, we used machine learning methods to screen out OS gene set A related to the occurrence and prognosis of AML and OS gene set B related to treatment in leukemia stem cells (LSCs) like population (HSC-like). Furthermore, we screened out the hub genes in the above two gene sets and used them to identify molecular subclasses and construct a model for predicting therapy response.

**Results:**

Leukemia cells have different OS functions compared to normal cells and significant OS functional changes before and after chemotherapy. Two different clusters in gene set A were identified, which showed different biological properties and clinical relevance. The sensitive model for predicting therapy response based on gene set B demonstrated predictive accuracy by ROC and internal validation.

**Conclusion:**

We combined scRNAseq and bulk RNAseq data to construct two different transcriptomic profiles to reveal the different roles of OS-related genes involved in AML oncogenesis and chemotherapy resistance, which might provide important insights into the mechanism of OS-related genes in the pathogenesis and drug resistance of AML.

## 1. Background

Oxidative stress (OS) is a series of adaptive responses caused by the imbalance between the reactive oxygen species (ROS) and the antioxidant system of all aerobic organisms. The integrated antioxidant system of an aerobic organism can block the damage caused by redundant ROS [1]. However, the abnormal redox state of cells can produce toxic effects of peroxides and free radicals, thereby damaging cellular proteins, lipids, and DNA, which can lead to aging, diseases, and tumorigenesis [2]. Some chronic diseases and cancers have been reported as a consequence of oxidative stress [3–5], including hypertension, Alzheimer's dementia, diabetes, breast cancer, and renal cell carcinoma [6, 7], while ROS shows bidirectional effects in cancer cells. On one hand, the ROS initiates tumorigenesis and supports the proliferation of cancer cells promoting the progression of cancer. On the other hand, high levels of ROS are also toxic to cancer cells causing cell death, which has been applied as a mechanism of chemotherapy [8]. Recent study showed that the combination of retinoic acid, tunicamycin, and arsenic trioxide can cause OS-induced cell death in FLT3-ITD+ AML cell lines [9]. Interestingly, tumor cells can increase their antioxidant capacity to accommodate high ROS-mediated cell proliferation while avoiding apoptosis and senescence triggered by excessive ROS, which might explain the chemotherapy resistance in cancers [10].

AML, the most common type of leukemia in adults worldwide, is characterized by the infiltration of abnormally clonal and undifferentiated leukemia cells in bone marrow, blood, and other tissues [11]. The enormous cytogenetic and molecular contexts make it a highly heterogeneous disease with distinct biological process and prognosis. Although the advance in treatment has been achieved among younger patients with a cure rate of 35-40%, the emergence of drug resistance often leads to treatment failure and the prognosis of elderly patients remains dismal; most patients died within one year of diagnosis [12, 13]. Therefore, the development of new therapies based on the individual genomic landscape risk stratification and understanding the mechanisms underlying drug resistance in leukemia are supposed to improve the remission rate and overall survival of AML patients. Currently, OS has been reported playing a role in the development of several hematologic cancers, including AML, chronic myeloid leukemia (CML), and acute lymphoblastic leukemia (ALL) [14, 15]. CML cells showed a higher oxidative stress with significant lower SOD activity, which is correlated with the altered intracellular calcium homeostasis. The increased oxidative stress and decreased antioxidants have been reported in ALL and AML patients in a recent study [14]. Dong et al. [16] also constructed an effective risk model for predicting the prognosis of AML patients based on the OS-related genes. However, previous studies mainly focus on the level of OS state and the salient factors leading to tumorigenesis and progression of AML, nothing has been done to explore the dual roles and potential mechanism of OS in AML.

In our present work, we selected the OS-related genes, which might contribute to the carcinogenesis of AML through scRNAseq combined with bulk RNA sequencing (RNAseq) data, and then established a molecular phenotype based on the above genes, which could predict prognosis and provide different OS profiles of AML patients. In addition, we established an OS-related gene signature for predicting the chemotherapy response of AML patients. All these could provide more information and genomic evidence for individualized precision therapy.

## 2. Material and Methods

### 2.1. scRNAseq and Bulk RNAseq Data Obtaining

The paired bone marrow scRNAseq data and clinical information of 8 AML patients (AML314, AML371, AML997, AML707B, AML475, AML329, AML556, and AML722B) and 4 healthy donors (BM1, BM2, BM3, and BM4) were obtained from Gene Expression Omnibus (GEO) database (https://www.ncbi.nlm.nih.gov/geo/) using the accession number GSE11625. We used the UCSC Xena (https://xenabrowser.net/) to download the GDC TCGA Acute Myeloid Leukemia (LAML) transcription expression data (*n* = 151) and phenotype data. The bulk RNAseq data and clinical information of AML patients (GSE106291, *n* = 250) were also downloaded from the GEO database. 1399 oxidative stress-related genes were selected according to a previous study [17].

### 2.2. scRNAseq Data Processing

The scRNAseq data analysis was performed in R version 4.1.3 as follows: (1) Seurat R package [18] was used to convert scRNAseq data as a Seurat object, and “NormalizeData” were used to preprocess and standardize the data; (2) the top 2000 highly variable genes after quality control were selected by “FindVariableFeatures”; (3) principal component analysis (PCA) was performed based on the 2000 genes to analyze the scRNAseq data; (4) uniform manifold approximation and projection (UMAP) was applied to explore the scRNAseq data; (5) a total of 21 different cell types were defined according to the original data and reference [19]; and (6) the “FindMarkers” function was used to find all markers of different cell types with the criterion FDR < 0.05 and |log2FC| > 0.5.

### 2.3. ssGSEA Algorithm to Evaluate the Oxidative Stress Functions

GO BP terms “RESPONSE_TO_OXIDATIVE_STRESS” and “CELL_DEATH_IN_RESPONSE_TO_OXIDATIVE_STRESS” were downloaded from the MsigDB database (https://www.gsea-msigdb.org/gsea/index.jsp) to evaluate oxidative stress-related functions and the R package “GSVA” to score each cell with the single-sample GSEA (ssGSEA) algorithm, setting as abs.ranking = T and parallel.sz = 3. The higher the total score, the higher the gene expression of each sample.

### 2.4. Functional Enrichment and Pathway Analysis

Gene Ontology (GO), Kyoto Encyclopedia of Genes and Genomes (KEGG), and Gene Set Enrichment Analysis (GSEA) functional enrichment analyses were performed using the cluster profiler package [20]. Three categories were included in the GO enrichment analysis, i.e., biological process (BP), cellular component (CC), and molecular function (MF). Default parameters are selected as the setting of “enrichgo” function, “enrichkegg” function, and “GSEA” function.

### 2.5. Nonnegative Matrix Factorization (NMF) Algorithm to Identify Molecular Subclasses

First, the differential genes derived from normal cells and tumor cells were intersected with OS-related genes. The significant prognostic genes in LAML dataset were selected by univariate Cox regression. Then, we used unsupervised NMF to cluster the data by using the package “NMF,” with the standard “brunet” for 10 iterations. The cluster value *K* was set at 2 to 10, and the optimal number of clusters was based on the cophenetic correlation coefficient. The relationship among clusters, clinical variables, and hub genes gene expression was shown by heatmap with R package “ComplexHeatmap.”

### 2.6. Construction and Validation of OS-Related Gene Risk Model

To select hub genes based on the above candidate genes, LASSO regression (LR) was first used selecting the minimal lambda value. Bootstrap_multicox regression and risk regression model were built according to the multivariate analysis results, and the formula of the model was as follows:

(1) $$\begin{array}{c} \text{Risk score}=\sum \frac{\text{Coef }i*\text{Gene }i}{\text{SD bootstrap}}. \end{array}$$

LAML dataset was treated as a training set, and GSE106291 was treated as a validated set. Patients were classified into a high- or low-risk group according to the risk score. Overall survival was defined as the endpoint. The R package “glmnet,” “boot,” “survival,” “survminer,” and “ggplot2” were used.

### 2.7. Selection of OS-Related Genes for Chemotherapy-Sensitive Model

First, the differential genes derived from AML-BC and AML-AC of the HSC-like population were intersected with OS-related genes. Then, a support vector machine (SVM) with “AvgRank” is sorted by the average ranking of 10-folds; target genes are obtained according to the best point of accuracy and error rate value. Random forest (RF) is used to select top 20 genes according to importancescore > 2.25, and the parameters of LASSO regression (LR) are nlambda = 100, alpha = 1, and nfolds = 10 in the “cv.glmnet” function sets. Then, select coef according to lambda.min, and finally, filter out the target genes. The intersection of three machine learning genes is used for constructing the therapy response predictive model. After multivariate logistic regression model analysis, a model formula was set.

### 2.8. Construction and Validation of a Chemotherapy-Sensitive Model

GSE106291 was the training set, and 50% of the samples were selected as the internal validation set randomly. Patients were divided into two groups (high-risk vs. low-risk group) according to the sensitive score. The receiver operating characteristic (ROC) was used to evaluate the model's predictability.

### 2.9. Statistical Analysis

All statistical analyses were performed in R version 4.1.3. LASSO regression and Cox regression analyses were conducted by the “glmnet” packages. The Wilcoxon rank sum test was used to compare the two groups. Contingency table variables were analyzed by chi-square test or Fisher's exact tests. Survival analysis was conducted by the KM method and compared by log-rank via “survival” packages. A two-tailed *P* value < 0.05 was indicative of a statistically significant difference.

## 3. Results

### 3.1. Single-Cell Profiling of AML Patients and Healthy Donors

**Figure 1:**
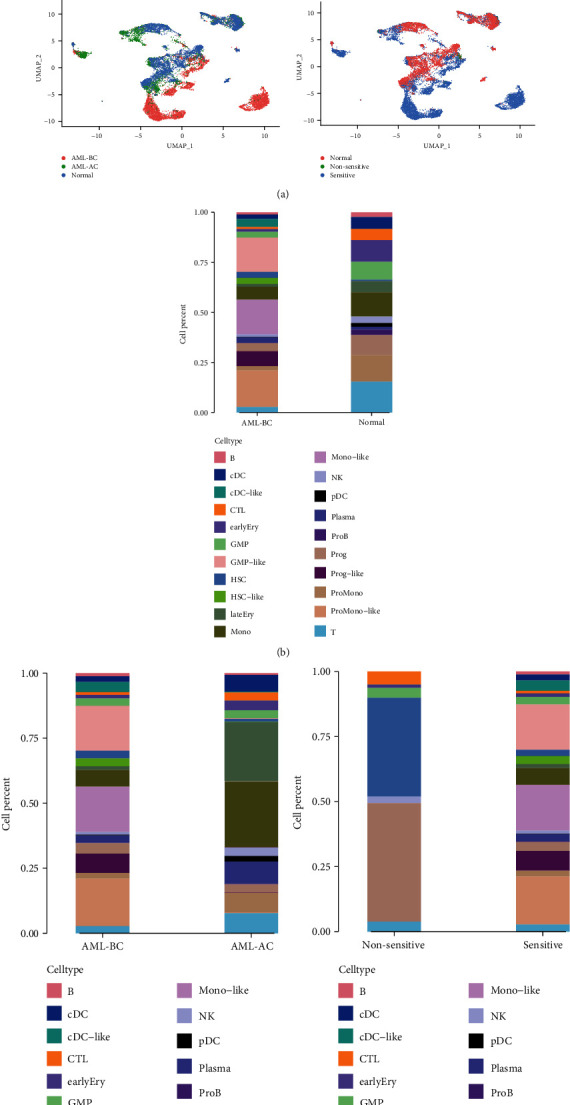
Single-cell profiling of AML patients and healthy donors. A total of 13593 cells and 21 cell types from 8 AML patients before and after induction chemotherapy and 4 healthy BM samples of GSE11625 are visualized by UMAP (a). Cell type composition in normal and AML patients before chemotherapy (b). Cell type composition in the AML-BC and AML-AC groups (c). Cell type composition in the sensitive and nonsensitive groups (d).

We integrated paired bone marrow scRNAseq data from 8 newly diagnosed AML patients before and after induction chemotherapy and 4 bone marrow samples from healthy donors. A total of 27899 features and13593 cells were included in this study. Among the 8 AML patients, AML 722B achieved morphological remission after two courses of induction chemotherapy and was included in the nonsensitive group; the left 7 AML patients achieved morphological remission after one standard course of chemotherapy, and they were included in the sensitive group. The cells of AML patients at initial diagnosis were classified as AML before chemotherapy (AML-BC) group, and the cells after induction chemotherapy were treated as AML after chemotherapy (AML-AC) group (Figure 1(a)). A total of 4625 malignant cells of six cell types (HSC-like, progenitor-like, GMP-like, promonocyte-like, monocyte-like, and cDC-like) were identified according to the defined cell types [19]. We can observe highly proliferative leukemia cells in AML patients and suppressed immune cells in the TME, compared with normal bone marrow samples (Figure 1(b)). Significant recovery of hematopoietic and immune cells was seen in these patients when they achieved morphological remission after induction chemotherapy (Figure 1(c)). Although fewer cells are in the nonsensitive group, differences in cell populations between the sensitive group and nonsensitive group can be observed (Figure 1(d)).

### 3.2. Differential Oxidative Stress Response in AML and Chemotherapy-Induced Changes in HSC-Like Subgroup

**Figure 2:**
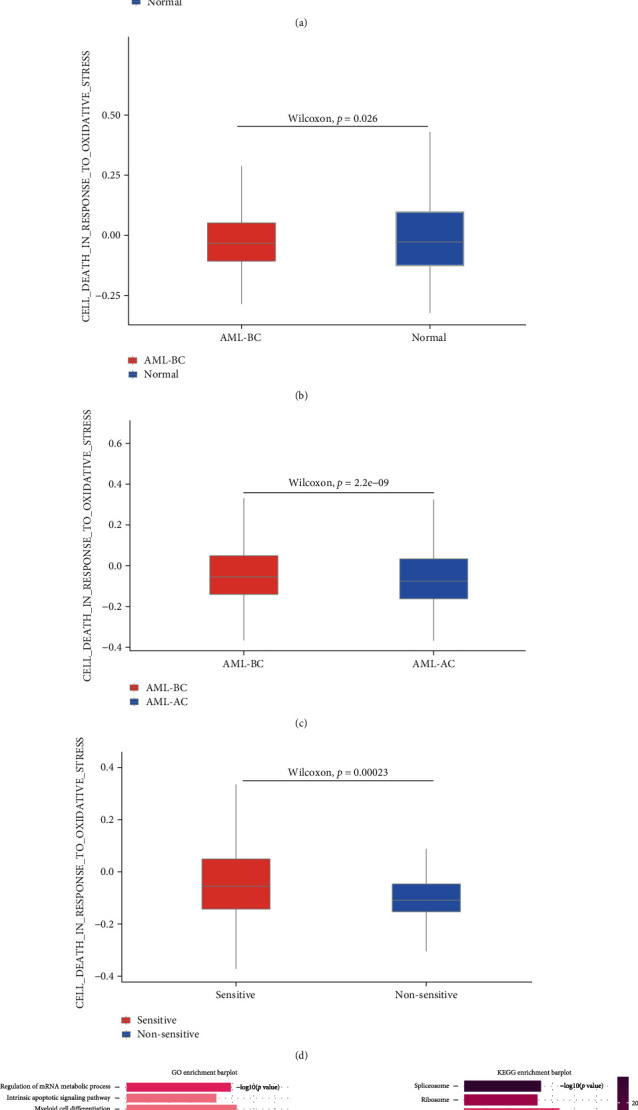
Differential oxidative stress functions in AML single cells. Normal cells have a higher response oxidative stress score (*P* = 0.003) and higher cell death in response to oxidative stress score than AML cells (*P* = 0.026) (a, b). Higher cell death in response to oxidative stress score in the AML-BC group than in the AML-AC group (*P* = 2.2*e* − 09) (c). Higher cell death in response to oxidative stress score in the sensitive group than the nonsensitive group (*P* = 0.00023) (d). GO and KEGG enrichment analyses of DEGs of the HSC-like subgroup pre- and postchemotherapy (e).

To assess oxidative stress in leukemia cells, we scored each cell in the AML-BC group and cells from normal bone marrow according to the relevant functional items. First, we evaluated the “GOBP_ RESPONSE_TO_OXIDATIVE_STRESS” and “GOBP_CELL_DEATH_IN_RESPONSE_TO_OXIDATIVE_STRESS” of the two types of samples. We found that normal cells have a higher response oxidative stress score (*P* = 0.003) and higher cell death in response to oxidative stress score than leukemia cells (*P* = 0.026) (Figures 2(a) and 2(b)). In addition, to find the subgroup most sensitive to oxidative stress caused by chemotherapy, we assessed the cell death in response to oxidative stress score of different leukemia subgroups before and after treatment. Although a significant difference in cell death in response to oxidative stress score of leukemia cells before and after chemotherapy was observed (*P* = 2.2*e* − 09) (Figure 2(c)), there was no significant difference in the scores of various leukemia cell subsets pre- and posttreatment (Supplementary Figure 1). Furthermore, the sensitive group also showed higher cell death in response to oxidative stress score than the nonsensitive group (*P* = 0.00023) (Figure 2(d)). Given that leukemia stem cells are the main source of drug resistance and disease progression, it is of great significance to explore the transcriptomic changes before and after treatment. We enriched the DEGs of the HSC-like subgroup pre- and posttreatment and found that these DEGs are mainly involved in the regulation of mRNA metabolic process, intrinsic apoptotic signaling pathway, myeloid cell differentiation, and homeostasis, along with the oxidative stress-related biological processes, and are mainly enriched in the spliceosome, ribosome, Parkinson's disease, pathways of neurodegeneration (multiple diseases), cellular senescence, and chemical carcinogenesis (reactive oxygen species pathways). GSEA also showed an activated OXIDATIVE_PHOSPHORYLATION pathway (*P* = 2*e* − 04) (Figure 2(e) and Supplementary Figure 2). These results implied that OS has taken part in the development of AML, and the elimination of leukemic stem cell populations is associated with chemotherapy-induced OS response.

### 3.3. Oncogenesis-Related OS Gene Set A in AML Single Cells

**Figure 3:**
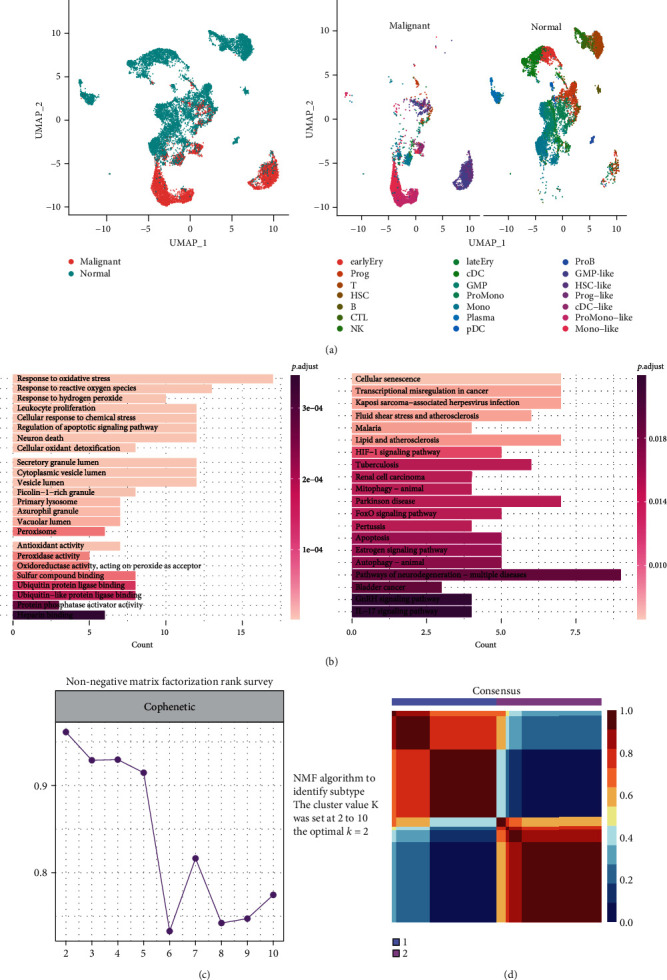
Identification of oncogenesis-related OS gene set A and function annotation. Cells of AML-BC and normal samples are visualized by UMAP (a). GO and KEGG enrichment analyses of oncogenesis-related OS gene set A (b). NMF to classify subclasses of OS gene set A in AML bulk RNAseq dataset LAML (c, d).

By intersecting the DEGs between initial leukemia cells and normal cells with OS-related genes, we screened out 59 AML oncogenesis-related OS genes, termed OS gene set A (Figure 3(a)) (Supplementary Table 1). The functional annotation also confirmed that these genes mainly take part in response to oxidative stress, response to reactive oxygen species, and some other redox-related pathways (Figure 3(b)).

### 3.4. Identification Subclasses of OS Gene Set A in AML Bulk RNAseq

**Figure 4:**
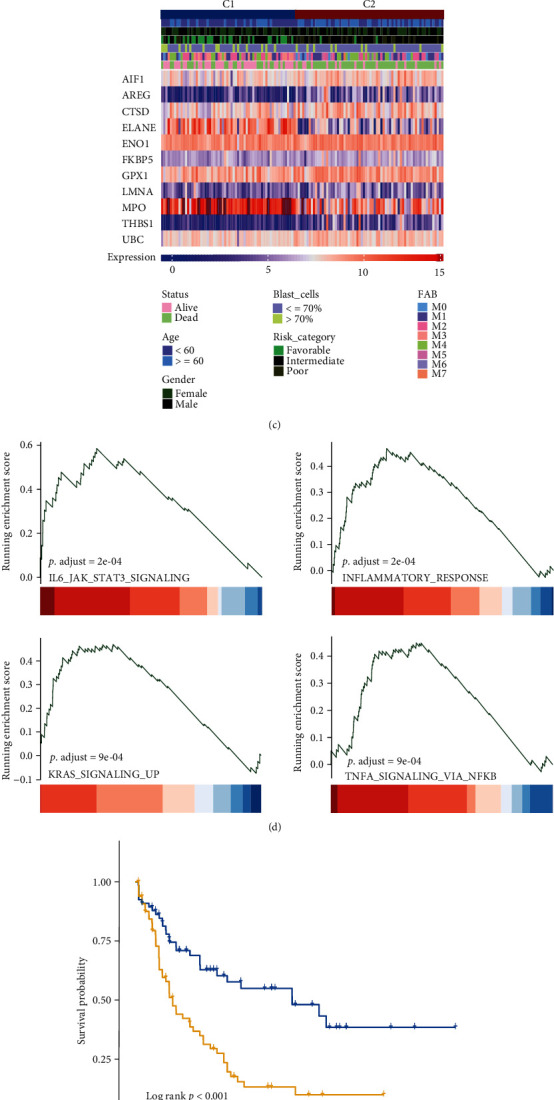
Different biological and clinical features of clusters in oncogenesis-related OS gene set A. Higher response to oxidative stress score by ssGSEA of C2 than C1 (*P* = 0.03) (a). AML patients in C2 have worse overall survival than C1 (*P* < 0.001) (b). The relationship between subclasses, clinical variables, and hub genes gene expression is visualized by heatmap (c). The significant enriched pathways of the two clusters by GSVA (d). AML patients in the high-risk score group have worse in LAML training dataset (*P* < 0.001) (e). AML patients in High-risk score group have worse in GSE106291 validation dataset (*P* < 0.001) (f).

To explore the possible mechanism of the above genes involved in AML, we used the LAML dataset as a training set to screen out 11 prognosis-related OS genes by Cox regression and classify them into 2 clusters (C1 and C2) according to the NMF cophenetic (Figures 3(c) and 3(d)). C2 showed a higher response to oxidative stress score than C1 (*P* = 0.03) (Figure 4(a)). Then, we further explore the relationship between the two clusters and the prognosis of AML, patients in C2 have worse overall survival than C1 (*P* < 0.001) (Figure 4(b)), and C2 also correlated with some clinical risk factors (age, risk category, and status) (Figure 4(c)) (Table 1). All of these suggest that these two clusters have different molecular and biological characteristics. The GSVA analysis of the two clusters group showed the activated INFLAMMATORY_RESPONSE pathway (*P* adjust = 2*e* − 04), IL6_JAK_STAT3_SIGNALING (*P* adjust = 2*e* − 04), KRAS_SIGNALING_UP (*P* adjust = 9*e* − 04), and TNFA_SIGNALING_VIA_NFKB (*P* adjust = 9*e* − 04) (Figure 4(d)). All these confirmed the oncogenic and prognostic roles of OS-related gene set A in AML.

### 3.5. Validation of the Prognostic Role of OS Gene Set A

To further validate the relationship between OS gene set A and prognosis, we used LASSO regression to get 6 hub genes (AIF1, ELANE, ENO1, GPX1, MPO, and THBS1) related to prognosis in the LAML dataset to construct a prognostic risk model (Supplementary Figure 3). Then, we got the risk model formula according to the coefficient of the bootstrap_multicox model.

(2) $$\begin{array}{c} \text{Risk score}=\sum \frac{\text{Coef }i*\text{Gene }i}{\text{SD bootstrap}}. \end{array}$$

AML patients are divided into a high-risk group and a low-risk group according to the median of each score. We found that patients have a different prognosis between the two risk groups (*P* < 0.001) (Figure 4(e)). At last, this survival difference was also validated in the GSE106291 dataset (*P* < 0.001) (Figure 4(f)).

### 3.6. Identification of Chemotherapy-Related OS Gene Set B in AML Single Cells

**Figure 5:**
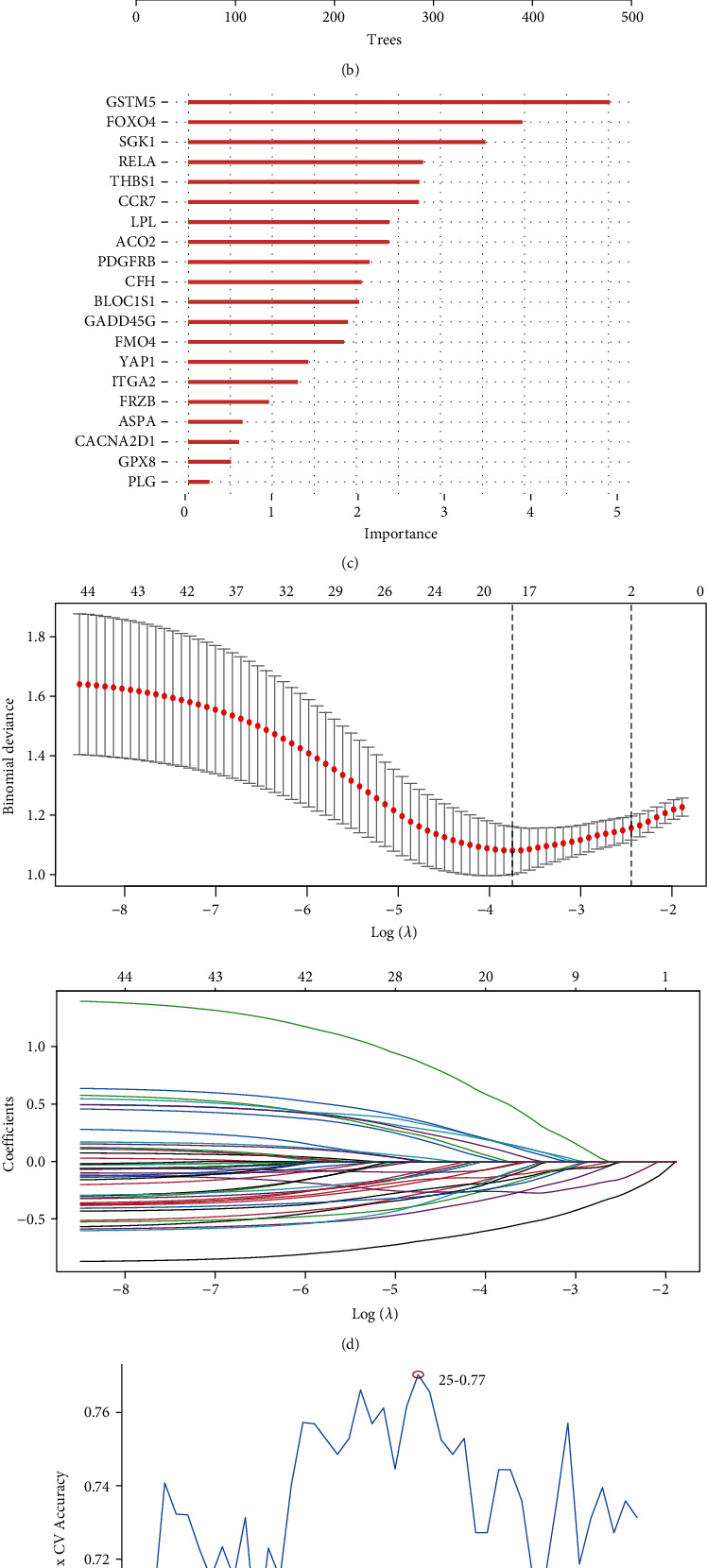
Selection of chemotherapy-related OS gene set B. Visualization of single cells in 21 subgroups of AML-BC and AML-AC (a). Random forest (RF), LASSO regression, and support vector machine (SVM) selected the chemotherapy-related OS gene set B (b–f).

To identify genes involved in chemotherapy-induced OS, we intersected the DEGs before and after chemotherapy of leukemia stem cell- (LSC-) like cells (HSC-like) with OS-related genes; 44 chemotherapy-related OS genes were screened out as OS gene set B (Figure 5(a)) (Supplementary Table 2).

### 3.7. Construct a Sensitive Model to Predict Therapy Response Based on OS Gene Set B in AML Bulk RNAseq

GSE106291 dataset was used as a training set. RF, SVM, and LASSO regression models were used to select model genes for predicting therapy response. We performed the RF algorithm to select a set of 20 candidate genes (Figures 5(b) and 5(c)), the LASSO algorithm to identify a set of 17 candidate genes (Figure 5(d)), and the SVM algorithm to select a set of 25 candidate genes (Figure 5(e)). At last, we choose the intersected 9 genes obtained by the above three machine learning algorithms to construct a model for predicting treatment response (Figure 5(f)). After multivariate logistic regression model analysis, we got a sensitive score for each patient according to the model formula.

### 3.8. Validation of the Predictive Role of OS Gene Set B

**Figure 6:**
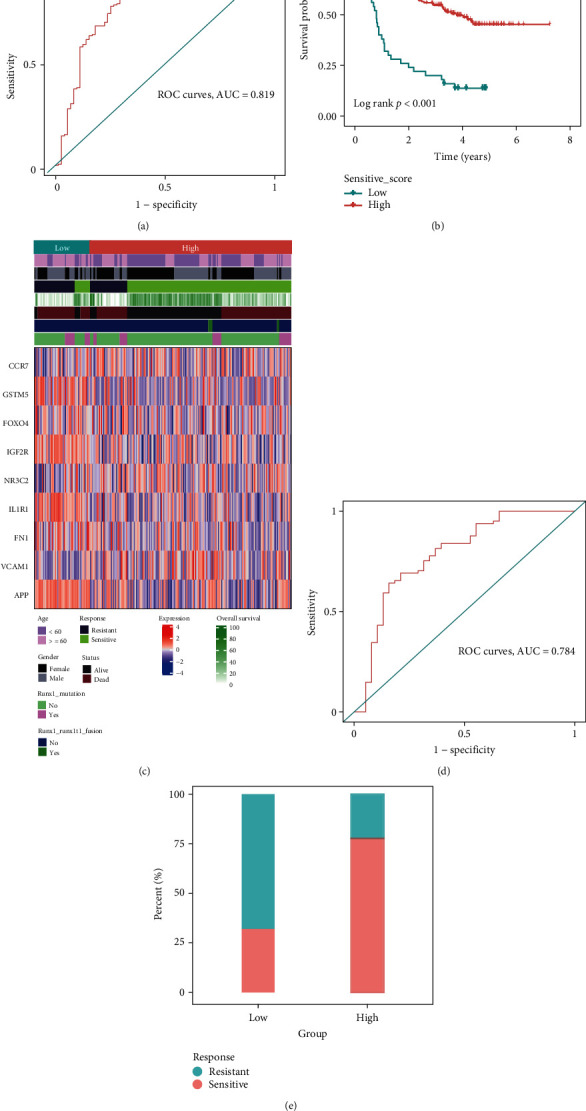
The predictive role of OS gene set B. ROC to evaluate the predictive accuracy of the training dataset GSE106291 (AUC = 0.819) (a). Patients in the high-sensitive group have better overall survival than patients in the low-sensitive group (*P* < 0.001) (b). The relationship between high- and low-sensitive groups, clinical variables, and hub genes gene expression are visualized by heatmap (c). ROC to evaluate the predictive accuracy of the internal validation of GSE106291 (AUC = 0.784) (d). Patients in the high-sensitive score group are more sensitive to chemotherapy (e).

Patients in the training set were divided into low- or high-sensitive groups based on a value of 0.5. We used ROC to evaluate the predictive accuracy of the model; the AUC of the training set is 0.819 (Figure 6(a)). In addition, we found that patients in the high-sensitive group have better overall survival than patients in the low-sensitive group (*P* < 0.001) (Figure 6(b)). Besides, some clinical variables, like age, gender, and life status, are correlated with the sensitive score (Figure 6(c)). After validation in the internal dataset, the AUC of the ROC in the validation dataset is 0.784 (Figure 6(d)), and patients in the high-sensitive score group are more sensitive to chemotherapy (Figure 6(e)). All these supported the effective predictive ability of the model.

## 4. Discussion

High levels of ROS can either lead to the death of normal cells through the process of programmed cell death or activate redox-sensitive transcription factors, like forkhead box class O (FoxO) transcription factor, which can regulate cell proliferation, apoptosis, and differentiation and lead to tumor formation and cancer progression [21]. As a clonally malignant disease, the role of OS in the pathogenesis of AML is not fully understood. Previous studies reported that the abnormal mutants in leukemia cells, like FIT3-ITD and Ras (N-Ras and H-Ras), could increase the DNA double-strand breaks and induce the production of superoxide, which lead to the occurrence of leukemia and promote the proliferation of these malignant cells [22, 23]. Conversely, alterations of redox homeostasis in both normal and oncogenic cells can lead to death. Studies have been initiated to explore redox-related mechanisms and proteins to potentially target leukemia cells. Cytarabine and azanucleoside DNA methyltransferase (DNMT) inhibitors are widely used in AML patients, which have been revealed to cause a substantial increase in ROS in both resting and leukemia cells and trigger cell cycle arrest and apoptosis [24, 25]. Therefore, improving the current understanding of the underlying mechanisms of OS generation in leukemogenesis and antioxidant therapy will facilitate the progress in AML risk stratification and therapeutic area.

Benefit from the bulk RNAseq and scRNAseq technology, we can interpret the genomic profile of disease at the single-cell level. The normal cells have more response to oxidative stress score and are more prone to oxidative stress-induced cell death than leukemia cell populations in our data, which was consistent with the high proliferation of tumor cells and the character that tumor cells can produce more peroxidative substances to escape the toxic effects of ROS by-products [26]. Although our results showed a significant difference of cell death in response to oxidative stress score between AML-BC and AML-AC groups, there is no significant difference in different leukemia cell subsets, which might be due to that the tumor microenvironment includes immune cells, stromal cells, and soluble molecules in addition to tumor cells, and the crosstalk network of these components may affect the cellular response to oxidative stress. In addition, a significant difference between the sensitive group and the resistant group can be observed, which also suggested that OS-induced cell death was related to chemotherapy resistance. The maintenance of the quiescent state of hematopoietic stem cells depends on the low production of ROS and high antioxidant defense, while leukemia stem cells are more sensitive to OS compared with normal hematopoietic stem cells [27, 28]. The function annotation results of different leukemia populations also provided evidence that HSC-like populations play an important role in the chemotherapeutic drug-induced OS. Given the bidirectional effect of ROS, we screened the oncogenesis-related OS gene set A and the chemotherapy-related OS gene set B from the scRNAseq data. Both two gene sets have biological processes, like response to oxidative stress, response to reactive oxygen species, and some other redox-related items by GO enrichment analysis. The results of KEGG also suggested that the oncogenesis-related gene set might involve cellular senescence, transcriptional misregulation in cancer, HIF-1 signaling pathway, and FoxO signaling pathway, which have been revealed by previous studies to be involved in the tumorigenesis of AML [29–32]. In addition, the PI3K-Akt signaling pathway and NF-kappa B signaling pathway were enriched by the OS gene set B. Growing evidence suggested that targeting PI3K-Akt signaling pathway may represent an effective treatment to kill AML LSCs [33, 34], and NF-kappa B inhibitor LC1 also showed an inhibitory effect on primary AML cells in vitro [35].

The two different clusters of prognosis-related OS genes displayed distinct molecular and clinical features. It seems that the higher the oxidative stress score, the worse the prognosis, and the higher oxidative stress score group was also related to some clinical risk factors, like age and risk category [36]. We found that these differential genes were mainly enriched in the INFLAMMATORY_RESPONSE pathway, IL6_JAK_STAT3_SIGNALING, KRAS_SIGNALING_UP, and TNFA_SIGNALING_VIA_NFKB through GSVA analysis, which might be important targets for future antiperoxidative therapy in AML. Notably, myeloperoxidase (MPO), as a common marker to distinguish between the myeloid and lymphoid lineages, was mainly expressed in the low oxidative stress score group according to our result. Previous contradictory study has addressed its clinical significance in pediatric B-acute lymphoblastic leukemia (B-ALL) patients [37], and Kuang et al. [38] identified MPO as a risk gene in AML patients. This contradictory result may be due to the discrepancy between individuals and the biological functions of MPO involved in different pathways. In addition, we constructed a nine-OS-related gene signature that can effectively predict the therapy response from the chemotherapy-related OS gene set B by different machine learning methods. Among these genes, C-C chemokine receptor type 7 (CCR7) encodes the protein CCR7, which is a member of the G protein-coupled receptor (GPR) family and expressed in various activated B and T lymphocytes, and has lower expression in nonresponders compared to responders involved in therapy resistance of AML which is revealed by a recent study [39]. Interleukin 1 receptor type I (IL1R1), also known as CD121a, is an important mediator involved in many cytokine-induced immune and inflammatory responses. Stratmann et al. [40] reported IL1R1 as a biomarker associated with AML progression and attenuated cellular growth and disease progression in primary AML cells, and AML murine models can be observed when suppressing IL-1 signaling [41]. All these suggest that the chemotherapy-related signature might involve the OS-induced immune response and inflammation to mediate chemotherapy resistance, and further stratified therapy could be adapted according to our signature.

In conclusion, our study is the first one to combine scRNAseq and bulk RNAseq data to construct two different transcriptomic profiles to reveal the different roles of OS-related genes involved in AML oncogenesis and chemotherapy resistance, which not only provide potential biological targets for the treatment of AML but also provide important insights into the mechanism of OS-related genes in the pathogenesis and drug resistance of AML. However, there are some limitations to our study. First, the occurrence of AML and drug resistance are closely related to the tumor microenvironment; our study mainly focused on the leukemia cell populations while not assessing the cell communication between leukemia cells and tumor-infiltrating immune cells mediated by the OS-related genes from the single-cell perspective. Second, more AML cohorts with scRNAseq data, which contain more patients with resistant information, should be included to reveal the role of OS-related genes in AML drug resistance. Third, further experiments should be done to explore the roles of hub OS genes in AML.

## Figures and Tables

**Table 1:** The different clinical characteristics between the two subtypes of OS gene set A.

Characteristics	C1	C2	*P* value
*Age (years)*			0.002
<60	45	31	
≥60	17	37	
*Gender*			0.313
Female	31	28	
Male	31	40	
*Risk category*			<0.001
Favorable	27	3	
Intermediate/normal	27	46	
Poor	8	19	
*Blast cells*			0.17
≤70%	47	58	
>70%	15	10	
*FAB*			<0.001
M0	2	10	
M1	14	17	
M2	17	14	
M3	14	0	
M4	13	14	
M5	2	10	
M6	0	2	
M7	0	1	
*Status*			<0.001
Alive	37	15	
Dead	25	53	

## Data Availability

All raw data included in the study are available in TCGA and GEO databases.

## Supplementary Materials

Supplementary 1 Supplementary Figure 1: cell death in response to oxidative stress score of different leukemia cells subsets between AML-BC and AML-AC: HSC-like (A), prog-like (B), GMP-like (C), ProMono-like (D), mono-like (E), and cDC-like (F).

Supplementary 2 Supplementary Figure 2: DEGs of AML-BC and AML-AC in HSC-like subset were enriched in OXIDATIVE_PHOSPHORYLATION pathway of by GSEA analysis.

Supplementary 3 Supplementary Figure 3: LASSO regression to get 6 hub genes related to prognosis in the LAML dataset to construct a prognostic risk model (A, B).

Supplementary 4 Supplementary Table 1: 59 AML oncogenesis-related OS genes.

Supplementary 5 Supplementary Table 2: 44 AML chemotherapy-related OS genes.
