# Supplementary figures and images for: Combined scRNAseq and Bulk RNAseq Analysis to Reveal the Dual Roles of Oxidative Stress-Related Genes in Acute Myeloid Leukemia
Source: Oxid Med Cell Longev. 2023 Feb 9;2023:5343746. doi: 10.1155/2023/5343746 (PMC9938912; doi:10.1155/2023/5343746)

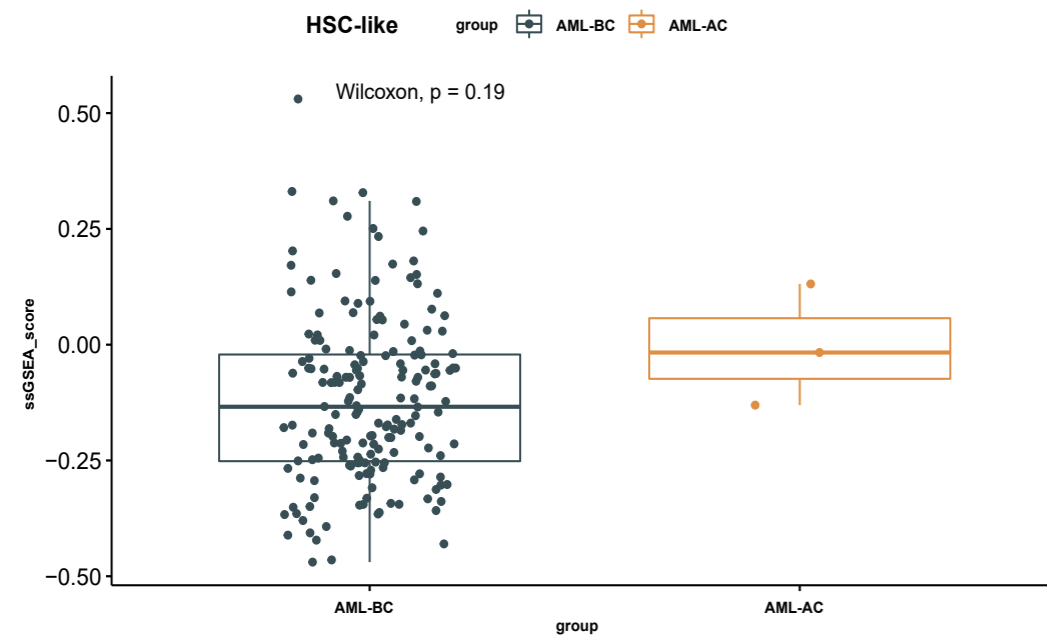

(A)

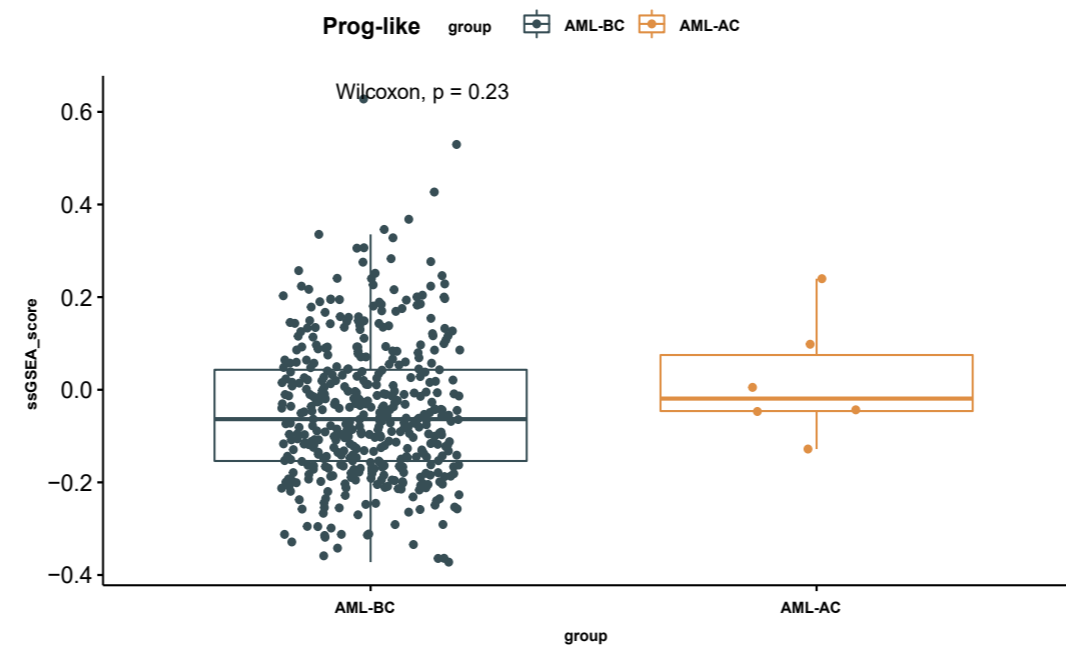

(B)

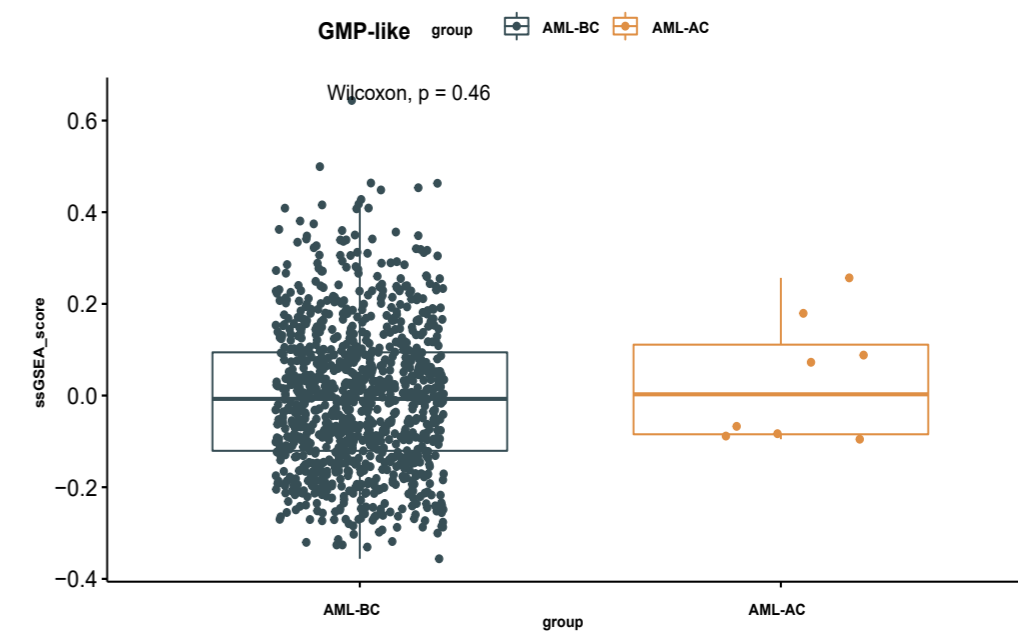

(C)

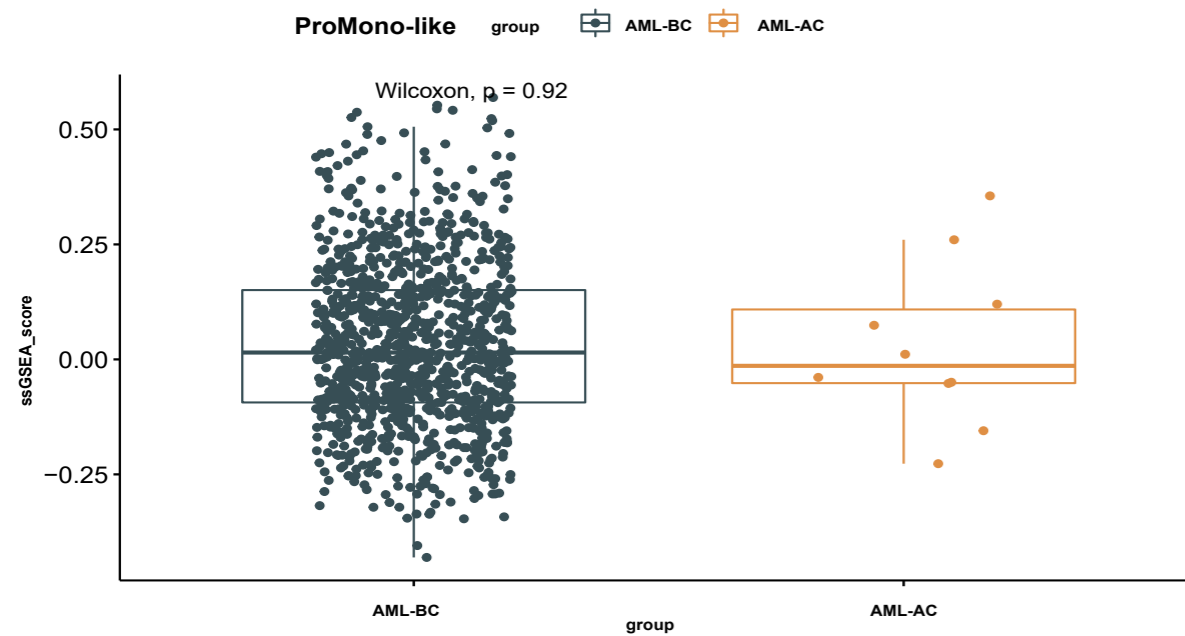

(D)

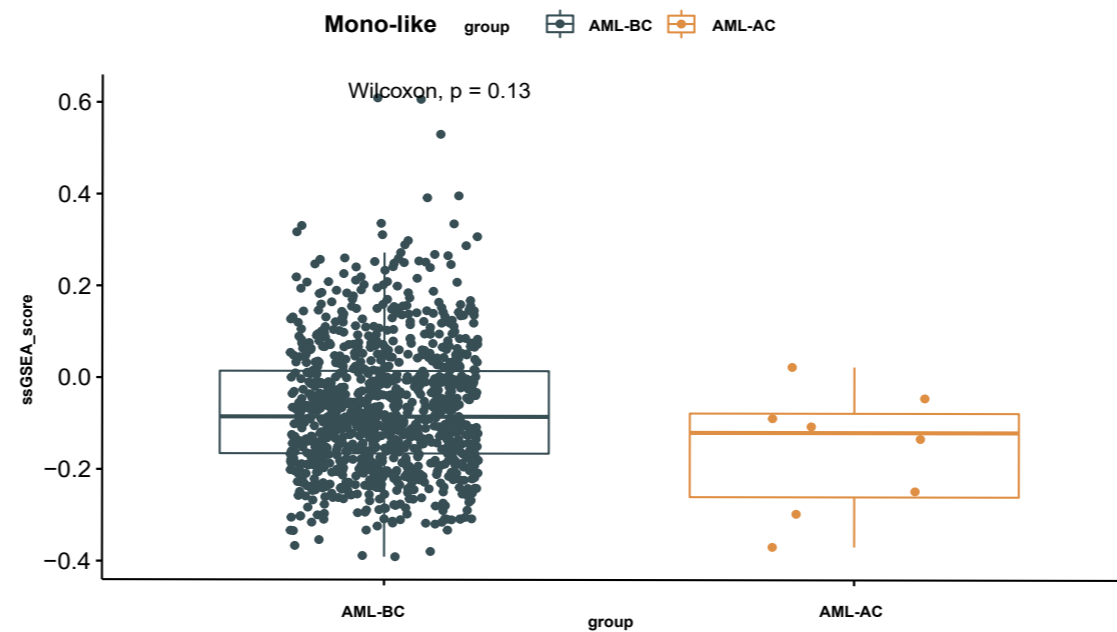

(E)

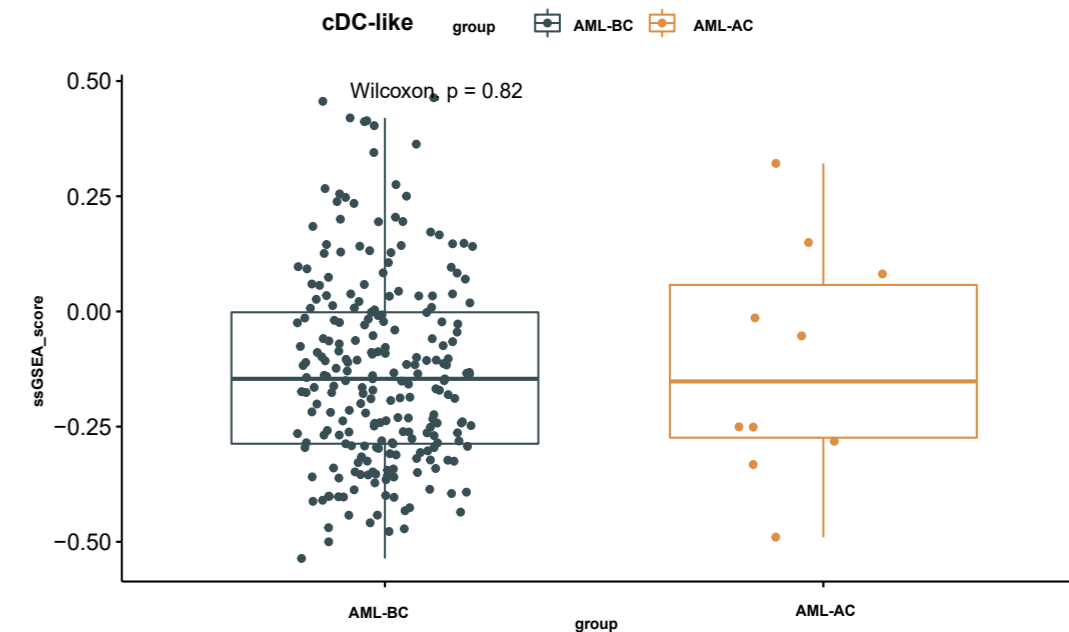

(F)

Supplement: Supplementary 1 — Supplementary Figure 1: cell death in response to oxidative stress score of different leukemia cells subsets between AML-BC and AML-AC: HSC-like (A), prog-like (B), GMP-like (C), ProMono-like (D), mono-like (E), and cDC-like (F). [file 5343746.f1.pdf]

# OXIDATIVE\_PHOSPHORYLATION

| pvalue | p.adjust |
|--------|----------|
| 2e-04  | 0.0014   |

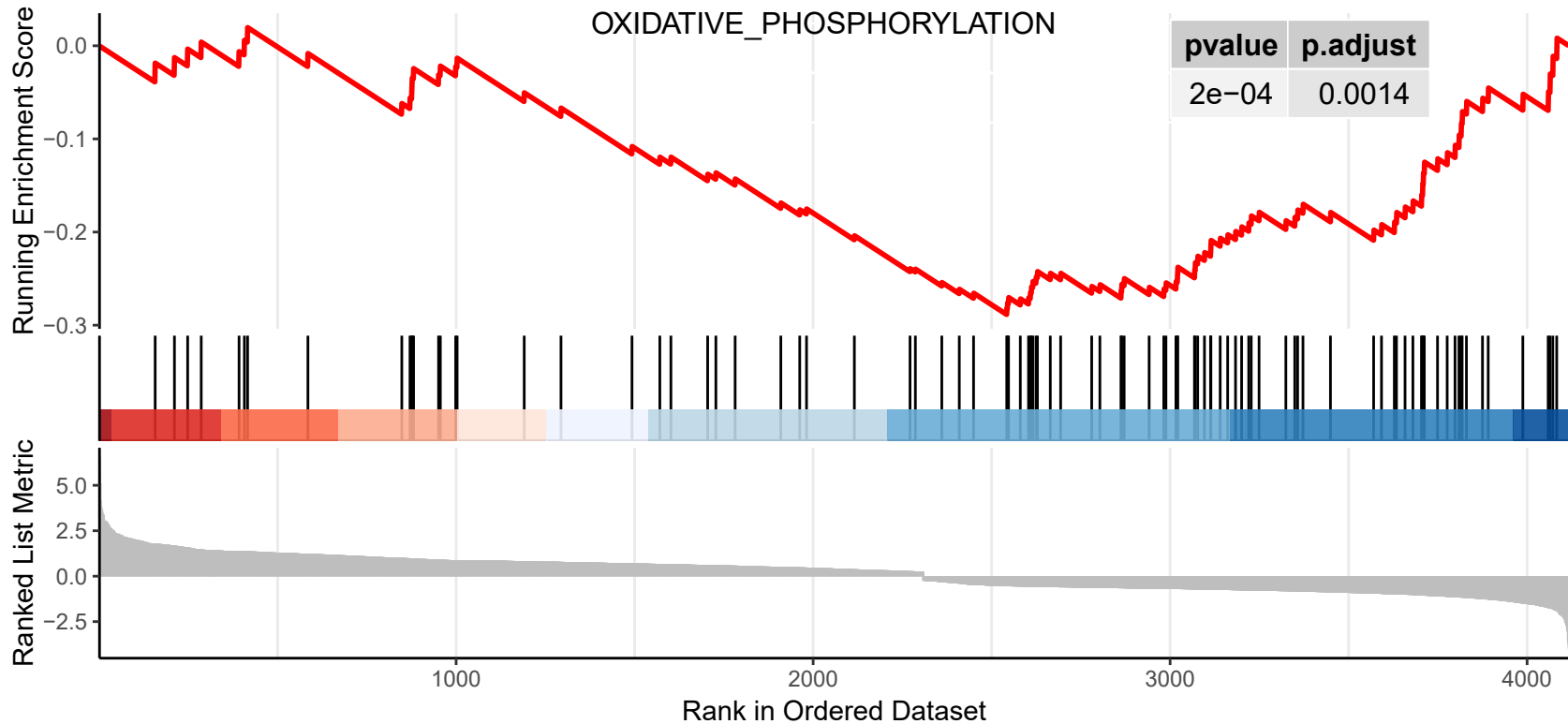

Supplement: Supplementary 2 — Supplementary Figure 2: DEGs of AML-BC and AML-AC in HSC-like subset were enriched in OXIDATIVE_PHOSPHORYLATION pathway of by GSEA analysis. [file 5343746.f2.pdf]

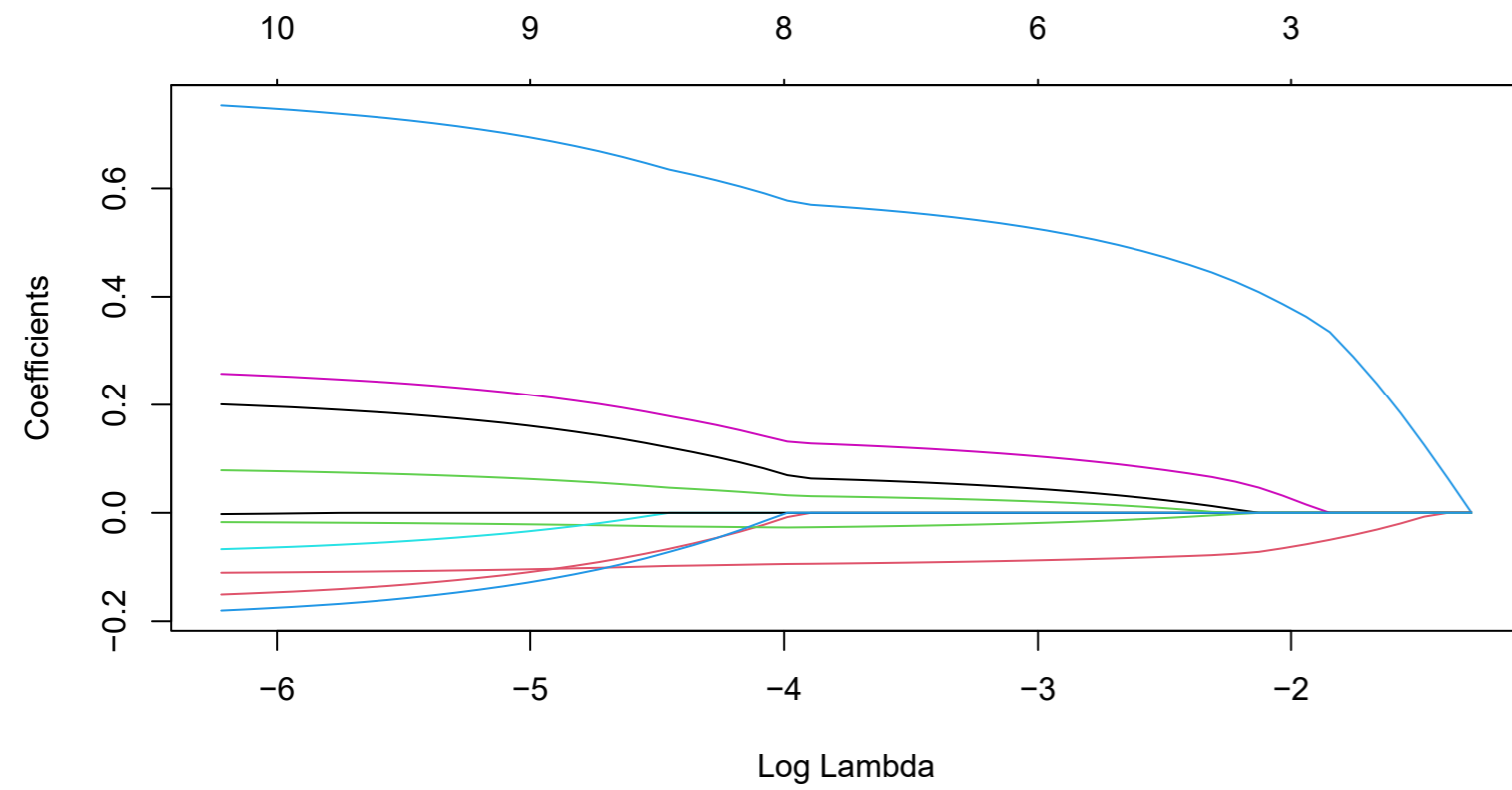

(A)

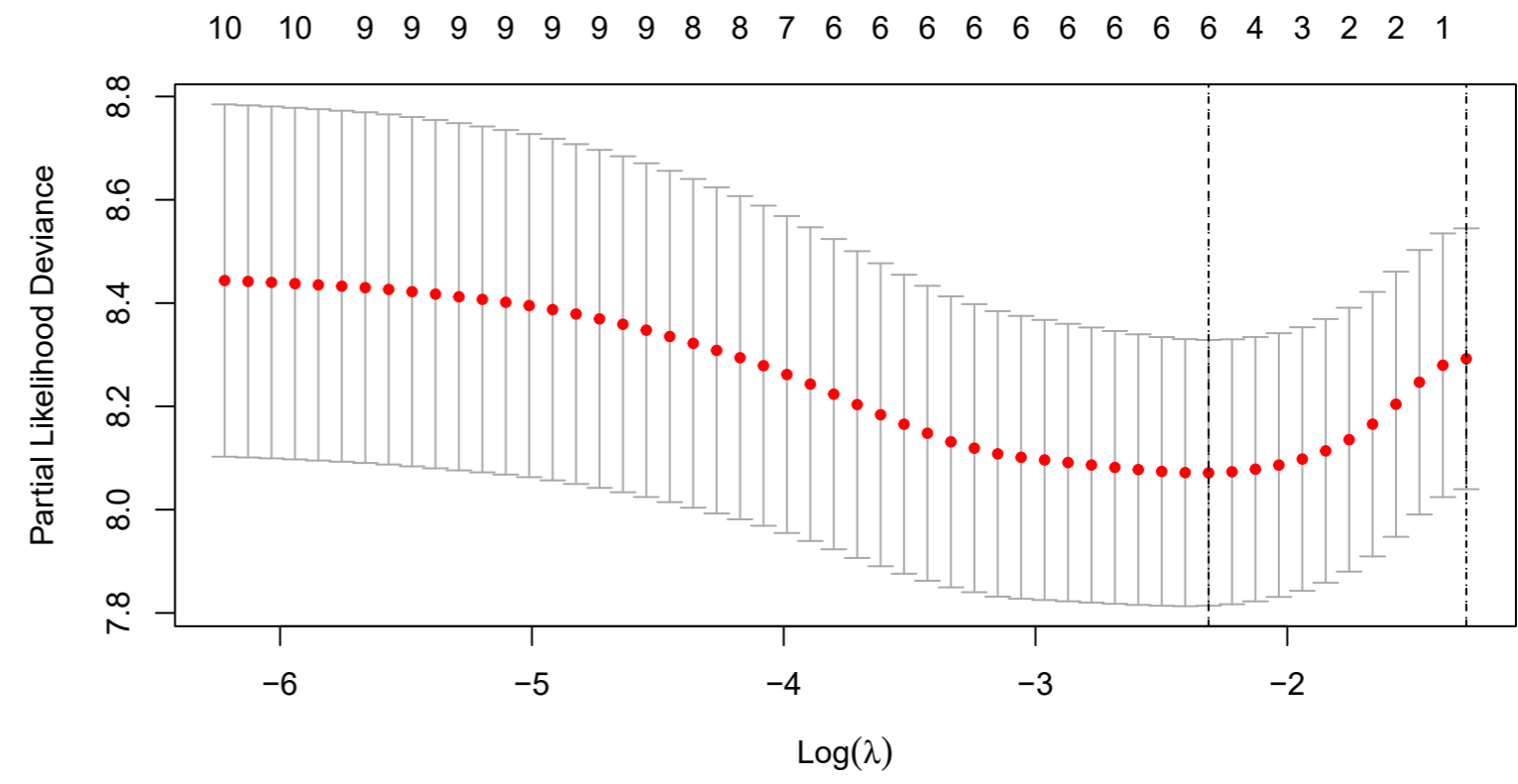

(B)

Supplement: Supplementary 3 — Supplementary Figure 3: LASSO regression to get 6 hub genes related to prognosis in the LAML dataset to construct a prognostic risk model (A, B). [file 5343746.f3.pdf]
