# Supplementary material for: Combined scRNAseq and Bulk RNAseq Analysis to Reveal the Dual Roles of Oxidative Stress-Related Genes in Acute Myeloid Leukemia
Source: Oxid Med Cell Longev. 2023 Feb 9;2023:5343746. doi: 10.1155/2023/5343746 (PMC9938912; doi:10.1155/2023/5343746)
Supplement: Supplementary 4 — Supplementary Table 1: 59 AML oncogenesis-related OS genes. [file 5343746.f4.pdf]

Sup table 1.AML oncogenesis-related OS genes

1	DHFR
2	AREG
3	PTPRC
4	MKI67
5	ATXN8OS
6	PSIP1
7	CR1
8	FTL
9	BCL6
10	UBC
11	SELL
12	CD34
13	CTSG
14	NEAT1
15	LGALS3
16	MT-ND6
17	FKBP5
18	MSRB3
19	THBS1
20	GRN
21	PPP1R15A
22	SGK1
23	AIF1
24	CALM2
25	MALAT1
26	AHSP
27	S100A9
28	VIM
29	TNFAIP3
30	ENO1
31	LAMP2
32	SERPINA1
33	CTSD
34	TFRC
35	EGR1
36	ETS1
37	ELANE
38	MT-ND5
39	DUSP1
40	MGST1
41	HSPD1
42	ATM
43	CDKN1A
44	HMGB1
45	CLU
46	HBB
47	CALM1
48	HIF1A
49	LMNA
50	DDIT3
51	GPX1
52	SQSTM1
53	HSPA5
54	GAPDH
55	JUN
56	CXCL8
57	MPO

58 CAT
59 SOD1
