# Supplementary material for: Combined scRNAseq and Bulk RNAseq Analysis to Reveal the Dual Roles of Oxidative Stress-Related Genes in Acute Myeloid Leukemia
Source: Oxid Med Cell Longev. 2023 Feb 9;2023:5343746. doi: 10.1155/2023/5343746 (PMC9938912; doi:10.1155/2023/5343746)
Supplement: Supplementary 5 — Supplementary Table 2: 44 AML chemotherapy-related OS genes. [file 5343746.f5.pdf]

Sup table 2. chemotherapy-related OS genes

1	GPX8
2	GADD45G
3	FMO4
4	ASPA
5	CCR7
6	FRZB
7	CACNA2D1
8	YAP1
9	ITGA2
10	ACO2
11	BLOC1S1
12	GSTM5
13	PDGFRB
14	FOXO4
15	LPL
16	CFH
17	PLG
18	THBS1
19	SGK1
20	RELA
21	TJP1
22	PDGFRL
23	ALDH1A1
24	EPAS1
25	CXCL12
26	IGF2R
27	ADH1C
28	PLA2G6
29	NR3C2
30	IL1R1
31	CD40
32	SGCB
33	ETS1
34	FN1
35	CYP1B1
36	PRKAA2
37	ANXA5
38	MMP2
39	VCAM1
40	APOE
41	CP
42	CCL2
43	APP
44	NOS1
